# Gapless quantum spin liquid ground state in the two-dimensional spin-1/2 triangular antiferromagnet YbMgGaO_4_

**DOI:** 10.1038/srep16419

**Published:** 2015-11-10

**Authors:** Yuesheng Li, Haijun Liao, Zhen Zhang, Shiyan Li, Feng Jin, Langsheng Ling, Lei Zhang, Youming Zou, Li Pi, Zhaorong Yang, Junfeng Wang, Zhonghua Wu, Qingming Zhang

**Affiliations:** 1 Department of Physics, Renmin University of China, Beijing 100872, P. R. China; 2 Institute of Physics, Chinese Academy of Sciences, Beijing 100190, P. R. China; 3 State Key Laboratory of Surface Physics, Department of Physics, and Laboratory of Advanced Materials, Fudan University, Shanghai 200433, P. R. China; 4 High Magnetic Field Laboratory, Chinese Academy of Sciences, Hefei 230031, P. R. China; 5 Institute of Solid State Physics, Chinese Academy of Sciences, Hefei 230031, P. R. China; 6 Wuhan National High Magnetic Field Center, Wuhan 430074, P. R. China; 7 Institute of High Energy Physics, Chinese Academy of Science, Beijing 100049, P. R. China; 8 Department of Physics and Astronomy, Collaborative Innovation Center of Advanced Microstructures, Shanghai Jiao Tong University, Shanghai 200240, P. R. China

## Abstract

Quantum spin liquid (QSL) is a novel state of matter which refuses the conventional spin freezing even at 0 K. Experimentally searching for the structurally perfect candidates is a big challenge in condensed matter physics. Here we report the successful synthesis of a new spin-1/2 triangular antiferromagnet YbMgGaO_4_ with 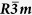 sy*m*metry. The compound with an ideal two-dimensional and spatial isotropic magnetic triangular-lattice has no site-mixing magnetic defects and no antisymmetric Dzyaloshinsky-Moriya (DM) interactions. No spin freezing down to 60 mK (despite θ_w_ ~ −4 K), the power-law temperature dependence of heat capacity and nonzero susceptibility at low temperatures suggest that YbMgGaO_4_ is a promising gapless (≤|θ_w_|/100) QSL candidate. The residual spin entropy, which is accurately determined with a non-magnetic reference LuMgGaO_4_, approaches zero (<0.6%). This indicates that the possible QSL ground state (GS) of the frustrated spin system has been experimentally achieved at the lowest measurement temperatures.

Low-spin geometrically frustrated systems in two-dimensional (2D) lattices have received significant interest in condensed-matter physics. The two most studied frustrated spin systems are spin-1/2 triangular and kagomé antiferromagnets, in which strong quantum fluctuations prevent spin freezing even at very low temperatures^1^. With respect to theoretical studies, Anderson first proposed that the triangular Heisenberg antiferromagnet (THAF) has a resonating valence bond GS, which is a type of spin liquid^2^,^3^. However, recent numerical studies have consistently indicated a long-range Néel GS for spin-1/2 THAF. The calculated order parameter is much smaller than the classical value, indicating that it is very close to a quantum critical point between magnetic ordered and disordered GSs^4^,^5^. On the experimental side, “structurally perfect” triangular or kagomé antiferromagnets (AFs) are still extremely rare, although many spin-1/2 geometrically frustrated triangular and kagomé AFs have been proposed^6^,^7^,^8^,^9^,^10^,^11^,^12^. Most of the existing candidates suffer from spatially anisotropic intralayer exchange interactions^6^,^7^,^9^,^12^, site-mixing between magnetic and nonmagnetic ions^12^,^13^,^14^, interlayer exchange interactions^8^,^9^,^10^, and/or antisymmetric Dzyaloshinsky-Moriya (DM) interactions^15^. These factors are critical to the GSs of the frustrated spin systems and are difficult to determine precisely. The complications caused by these factors make extraction of the intrinsic physics from real systems difficult.

In this paper, we report the successful synthesis of a new triangular antiferromagnet with effective spin-1/2, YbMgGaO_4_. The aforementioned structural disadvantages are avoided in the new compound. First, it has spatially isotropic and perfect triangular layers with 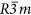 symmetry. Second, the number of magnetic defects is negligible because of the large chemical difference between the Kramers magnetic Yb^3+^ ions and the nonmagnetic ions. Third, the magnetic triangular layers are well magnetically separated by nonmagnetic double layers of Mg/GaO_5_ triangular bipyramids, indicating an ideal two-dimensionality of the magnetic layers and a negligible interlayer exchange interaction. Finally, the antisymmetric DM interactions between first-, second- and third-neighbor spins are strictly excluded because inversion centers are located at any Yb^3+^ ion and at the half-way sites between them^16^. Moreover, the nonmagnetic reference compound LuMgGaO_4_ is also available for control experiments, such as precisely excluding the lattice heat capacities for YbMgGaO_4_.

No magnetic ordering is observed at least down to 60 mK from both magnetization and heat capacity measurements, despite the obvious AF exchange interaction between nearest-neighbor spins (θ_w_ ~ −4 K) suggesting that YbMgGaO_4_ is a new QSL candidate. And the power-law temperature dependence of heat capacity and nonzero susceptibility further indicate that the excitation gap from the GS should be no more than ~ |θ_w_|/100 at low temperatures^17^,^18^. Almost zero residual spin entropies are observed under 0 to 9 T at *T* < 0.3 K. This experimentally indicates that the frustrated spin system extremely approaches a possible gapless QSL GS at low temperatures. Moreover, an anomalous susceptibility plateau is first observed at paramagnetic states (0.5 K), which imply an unusual field-induced quantum spin state.

## Results and Discussion

YbMgGaO_4_ and LuMgGaO_4_ are members of the Ln^3+^M^2+^M′^3+^O_4_ family 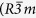 symmetry, *a* ~ 3.4 Å and *c* ~ 25 Å), where Ln is Lu or Yb, and M and M′ are 3d transition metals. Spin frustration has been observed in the other members of this structural family, including YbCuGaO_4_, LuCuGaO_4_, LuCoGaO_4_, LuZnFeO_4_ and LuCuFeO_4_^19^. Unfortunately, the chemical disorder in these compounds, along with the geometrical frustration, resulted in spin-glass behavior^19^. Compounds with magnetic ions occupying only the lanthanide sub-lattice (triangular layer), such as YbZnGaO_4_, have not been reported thus far. Our experiments demonstrate that pure YbZnGaO_4_ is difficult to synthesize in air because ZnO is volatile at *T* > 1200 °C. However, MgO remains stable at *T* < 1500 °C, as confirmed by the almost zero mass loss of this compound after heating. This thermal stability of MgO allows us to successfully synthesize pure YbMgGaO_4_.

**Figure 1:**
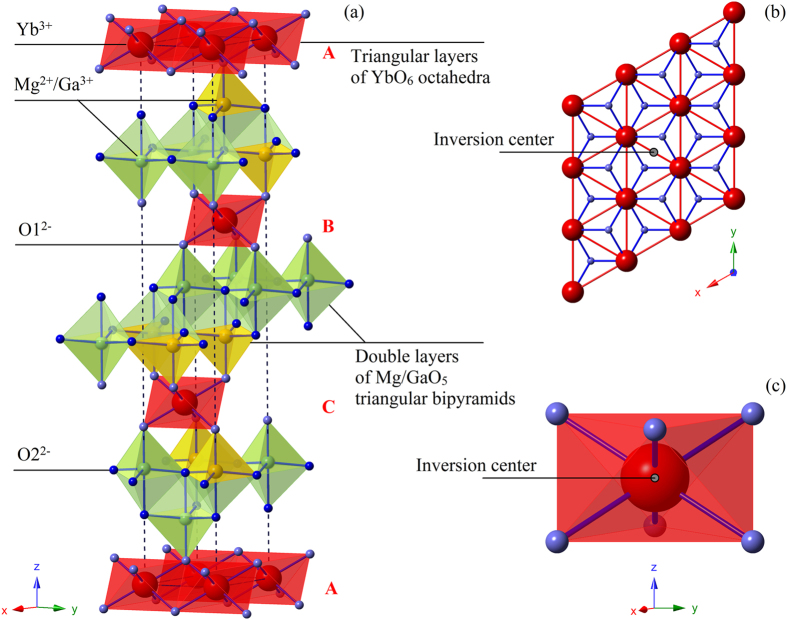
(**a**) Polyhedral structure of YbMgGaO_4_. The black dashed lines indicate the unit cell. (**b**) Top view of the triangular layer of YbO_6_ octahedra. (**c**) Local crystal structure around the Yb^3+^ Kramers ions.

For accuracy, we employed two starting crystal structure models: YbFe_2_O_4_ (I) and LuCuGaO_4_ or YbCoGaO_4_ (II)^19^. In model I, Ln^3+^ locates at its ideal position (0, 0, 0). We fit the observed XRD intensities with reasonable refined parameters and small residuals in the Supplementary Information. In the case of the alternative model II, Ln^3+^ slightly deviates from its ideal position to (0, 0, *z*_Ln_), where *z*_Ln_ ~ 0.004. In this case, we also achieved a reasonable refinement using model II. However, an extra refinement parameter *z*_Ln_ was required. Even when the deviation exists, the maximum displacement along the *c*-axis is only approximately ± 0.004*c* (0.1 Å). This possible displacement may be due to Mg^2+^-Ga^3+^ disorder in the interleaved double layers (Fig. 1a) and may cause slight bond randomness. Other researchers have reported that *z*_Ln_ is ~ 0.009 in LnCuGaO_4_, whereas *z*_Lu_ is ~ 0.006 in LuCoGaO_4_ and *z*_Yb_ is ~ 0.005 in YbCoGaO_4_^19^,^20^. These results are consistent with the fact that Cu^2+^ is Jahn-Teller active, whereas Mg^2+^, Co^2+^ and Ga^3+^ are not. Notably, the strict 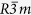 symmetry is maintained in both cases; i. e., a triangular spin lattice remains spatially isotropic.

The crystal structure is shown in Fig. 1a. Triangular layers of magnetic YbO_6_ octahedra, interleaved with double layers of nonmagnetic Mg/GaO_5_ triangular bipyramids, are ABC-stacked along the *c*-axis. Thus, the interlayer magnetic coupling can be negligible compared to the intralayer superexchange interaction between neighboring Yb^3+^, which is directly mediated by two parallel anions, O1^2−^. Site-mixing between magnetic and nonmagnetic ions is forbidden because the radius of Yb^3+^ is much larger than that of Mg^2+^ or Ga^3+^. This result is confirmed by the lack of observable narrow electron spin resonance (ESR) signals of isolated Yb^3+^ ions in YbMgGaO_4_ (Supplementary Information). The spatially isotropic triangular layer of YbO_6_ is shown in Fig. 1b. Because the inversion centers are located at halfway sites between neighboring Yb^3+^ ions (Fig. 1b) and Yb^3+^ sites (Fig. 1c), the DM interactions between the first-, second-, and third- neighbor Yb^3+^ vanish according to Moriya’s rules^16^. Since Yb^3+^ is a Kramers ion, the low-T (≤30 K, see below) magnetism of YbMgGaO_4_ should be dominated by the Kramers doublet GSs with effective spin-1/2.

**Figure 2:**
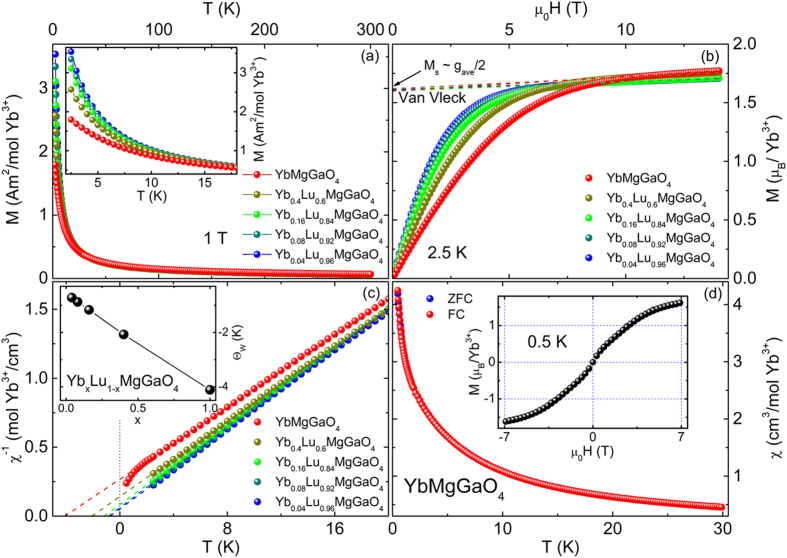
(**a**) Temperature dependence of magnetization under zero-field-cooling (ZFC) and 1 T in Yb_*x*_Lu_*1–x*_MgGaO_4_. Inset: zoomed view of the low-temperature data. (**b**) Magnetic field dependence of magnetization at 2.5 K. The colored dash lines show Van Vleck paramagnetism extracted from the linear-field-dependent magnetization data (>10 T). (**c**) Curie-Weiss fits of magnetization data at low temperatures (<20 K). Inset: fitted (AF) Weiss temperatures. (**d**) Susceptibilities measured under ZFC and FC from 0.48 to 30 K. No splitting between the ZFC and the FC data was observed at temperatures above 0.48 K. Both the cooling field H_c_ and the measurement field H_m_ are 100 Oe. Inset: complete magnetic loop measured at 0.5 K. In both the first and third quadrants, the data collected under increasing fields are perfectly overlapped by those collected during decreasing field.

The magnetizations are strongly suppressed with increasing Yb^3+^ concentration or effective exchange coupling (Fig. 2a,b); this result suggests an AF neighbor exchange energy. The AF coupling is confirmed by the negative Weiss temperatures, θ_w_ < 0 (Fig. 2c). The Curie-Weiss fit gives the Weiss temperature, θ_w_ = −4.11(2) K, and the averaged g-factor, 3.2(1), for YbMgGaO_4_. At T ≪ |θ_w_|, there is a slight deviation from the Curie-Weiss law (Fig. 2c), which corresponds to an upturn of susceptibility (Fig. 2d). A broad peak in bulk susceptibility well below |θ_w_|, which was observed in lots of frustrated spin systems^6^,^7^, is absent in our samples. The underlying reason why the broad peak is absent in YbMgGaO_4_ is still unclear at present, considering the fact that impurity spins can be ignored and DM interactions are symmetrically forbidden in YbMgGaO_4_ (Fig. 1b,c). To reveal the exact reason, further experimental and theoretical efforts based on high-quality single crystals^21^ are highly required. In fact, the broad peak seems not to be a common feature of 2D frustrated spin systems. For instance, it is absent in the bulk susceptibility of herbertsmithite no matter whether the contribution from impurity spins is taken into account or not^22^. The spin-1/2 antiferromagnetic Heisenberg model on the triangular lattice with a quenched randomness^23^ (∆ > 0.7) or on the kagome lattice with DM interactions^24^ (D_p_ ~ 0.3 J) also gives no broad peak in uniform susceptibility well below |θ_w_|. Saturation behavior is observed in the MH curves below 10 T (Fig. 2b), whereas a linear dependence emerges above 10 T; this behavior is attributed to Van Vleck magnetism^8^ and is related to field-induced electronic transitions. The saturation magnetizations are in agreement with the above averaged g-factor, M_s_ = 1.600(2) μ_B_/Yb^3+^ ~ g_ave_/2, where the powder-averaged g-factor, g_ave_ ~ 2/3 g_⊥_+1/3 g_∥_. In addition, a Van Vleck susceptibility (the fitted slope), χ_vv_ = 0.0122(1) μ_B_/Yb^3+^/T, is also obtained.

**Figure 3:**
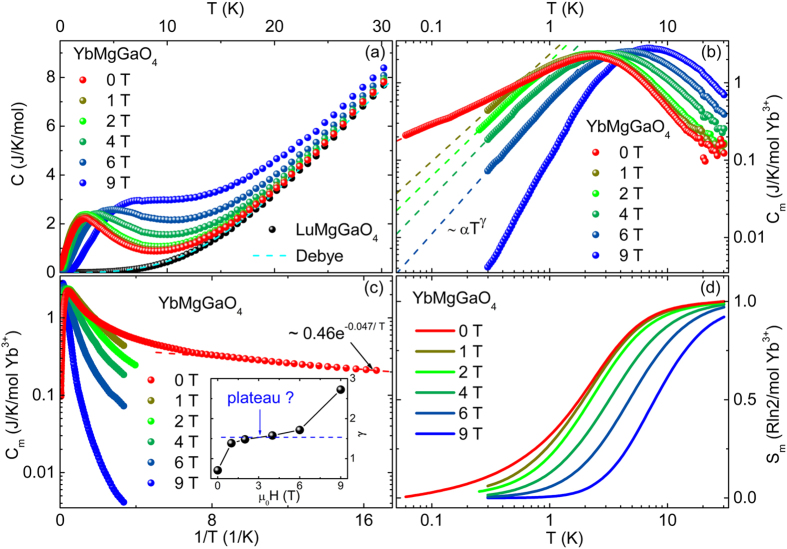
(**a**) Temperature dependences of total heat capacity under different magnetic fields in YbMgGaO_4_ and LuMgGaO_4_. The dashed curve denotes the Debye heat capacity. (**b**) Temperature dependences of magnetic heat capacity under different magnetic fields in YbMgGaO_4_. The colored dash lines show the power law fits to the low-*T* magnetic heat capacities. (**c**) Magnetic heat capacity vs. 1/*T* in YbMgGaO_4_. The red dash line shows the gapped spectral function fit to the low-*T* magnetic heat capacities under 0 T. Inset: fitted power exponent γ. (**d**) Temperature dependences of integral magnetic entropy under different magnetic fields in YbMgGaO_4_.

Experimentally determining the GS of YbMgGaO_4_ is of particular interest and non-trivial. The susceptibilities measured under zero field cooling (ZFC) and FC show no observable differences at least down to 0.48 K (Fig. 2d). A complete magnetization loop (−7 T to 7 T) was also measured at 0.5 K, and no hysteresis was observed (inset of Fig. 2d). The heat capacities measured under 0 to 9 T show no sharp λ-type peak down to 60 mK (Fig. 3a,b). These observations are incompatible with the long-range AF/ferromagnetic or short-range spin glass^25^/spin ice^26^ transition. It should be pointed out that the quasi-long-range Kosterliz-Thouless (K-T) transition also seems unlikely, since the above results are contrary to the typical thermodynamic changes accompanying the K-T transition^27^,^28^. The results consistently suggest no spin freezing at least down to 60 mK, despite the AF exchange energy (θ_w_ ~ −4 K). In addition, YbMgGaO_4_ is a good insulator with a room temperature resistance greater than 20 MΩ. These results imply that the compound is a new QSL candidate.

The discrepancy between the experimental observations and numerical results^29^ of the spin-1/2 triangular isotropic Heisenberg model, XY model or more general XXZ model is attributed to the following possibilities. First, the effective-1/2 exchange matrix between neighbor Yb^3+^ may significantly deviate from the ideal Heisenberg, XY or XXZ model^30^, and may cause much stronger spin frustration and fluctuation at low temperatures. And we would like to discuss this in detail in the further experimental and theoretical works basing on high-quality single crystals^21^. Second, slight bond randomness may exist in YbMgGaO_4_ according to the crystal structure model II, which may influence the GS. The bond randomness can prevent spin ordering in THAF according to the exact diagonalization (ED) study^23^. Third, the next-nearest-neighbor and/or longer exchange interactions may stabilize a spin disordered GS against AF states^31^,^32^. Finally, the multiple-spin^33^/ring exchanges^34^ can also prevent Néel long-range ordering in a triangular lattice. Quantum fluctuations play an important role in the calculated ordered GS because of the geometrical frustration even in Heisenberg case^5^. This fact means that some seemingly small perturbations may be critical in the determination of the GS of the frustrated spin system at low temperatures.

The heat capacity is a highly sensitive probe for the low-energy excitations from the GS. The availability of a perfect nonmagnetic reference compound LuMgGaO_4_ enables an accurate exclusion of the lattice heat capacities without any fitting. Such advantage is absent in most of the existing QSL candidates. The measured heat capacities of LuMgGaO_4_ well follow the Debye law with a Debye temperature ~ 151 K (Fig. 3a). Thus the exact magnetic heat capacities of YbMgGaO_4_ can be precisely extracted by directly subtracting the lattice contributions, i.e., the heat capacities of LuMgGaO_4_, from those of YbMgGaO_4_ (Fig. 3b). The heat capacity measurements were performed up to 30 K, at which the spin-1/2 entropy has been fully released as *T* ≫ |θ_w_|, and the Yb^3+^ ions remain in the Kramers doublet GSs with effective spin-1/2 as C_m_(~30 K, 0 T) ~ 0 (Fig. 3b). As the temperature decreases, the magnetic heat capacities of YbMgGaO_4_ exhibit a broad hump, whose position is almost field-independent (μ_0_H ≤ 2 T), and shifts to a higher temperature with further increasing applied magnetic fields (μ_0_H ≥ 4 T). The broad hump centered at 2.4 K (under 0 T) may suggest a crossover into a QSL state^35^. At *T* < 2 K, the magnetic heat capacities well follow power-law temperature dependences down to the lowest measurement temperatures (Fig. 3b). The fitted power exponent, γ ~ 0.7, approaches the theoretical value of 2/3 reported in the THAF spin liquid with ring exchanges^34^. It is another evidence that YbMgGaO_4_ is a new strongly correlated QSL candidate. γ increases up to 2.7 under 9 T (the inset of Fig. 3c) possibly because of the gradually overcoming of the 2D quantum spin correlations. We have also tried to fit the low temperature heat capacities (60 to 83 mK, 0 T) with a gapped spectral function, C*exp[−ΔE/*T*]. The fitted energy gap, ΔE = 0.0469(1) K, is no more than ~ |θ_w_|/100 (Fig. 3c). Moreover, the susceptibilities (Fig. 2d) show no downward trend to zero down to 0.48 K, which is much lower than the hump temperature. These consistently suggest that YbMgGaO_4_ is a QSL candidate with an excitation gap no more than ~ |θ_w_|/100.

The released magnetic entropies from the lowest measurement temperature to 30 K are exactly ~ Rln2/mol Yb^3+^ in YbMgGaO_4_ (Fig. 3d). In other words, the residual spin entropy at 60 mK is almost zero. This means that the gapless QSL candidate has a disordered but macroscopically non-degenerate GS at low temperatures, and thus the third law of thermodynamics remains unviolated. Moreover, the near-zero upper limit of residual entropy (~0.6%) at 60 mK almost excludes the possibility of magnetic transitions at lower temperatures and indicates that a possible QSL GS is experimentally achieved in YbMgGaO_4_, since there are no sufficient entropies for spin symmetry breaking any more below 60 mK.

The neighbor exchange energy, |θ_w_|, is on the order of several Tesla. The magnetization of YbMgGaO_4_ under a stable magnetic field at very low temperatures is critical and interesting. Unconventional quantum spin states, such as quantum magnetization plateaus^8^,^9^,^10^, can be induced by applied fields.

**Figure 4:**
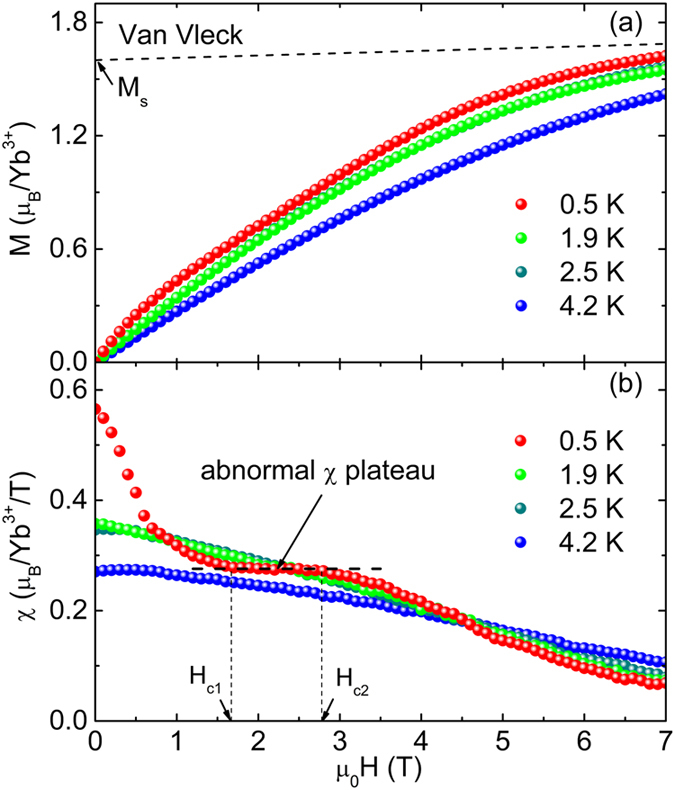
(**a**) Magnetic field dependence of magnetization at 0.5, 1.9, 2.5 and 4.2 K. The dashed line represents Van Vleck magnetism. (**b**) Magnetic field dependence of susceptibilities (dM/dH) at 0.5, 1.9, 2.5 and 4.2 K. The dashed lines show the interval where almost constant susceptibilities are observed.

The careful magnetization measurements of YbMgGaO_4_ at 0.5 K under 0 to 7 T, as well as at higher temperatures, are shown in Fig. 4a. The derived susceptibilities (dM/dH) show a clear and anomalous plateau under fields of 1.6 to 2.8 T at 0.5 K, and this plateau completely disappears at higher temperatures (1.9, 2.5 and 4.2 K), as shown in Fig. 4b. The unconventional magnetization behavior was confirmed by repeating the measurements several times. It is unlikely that the anomalous plateau originates from impurity spins. As aforementioned, the ESR measurements have determined a negligible amount (<0.04%) of impurity/isolated spins. This is further confirmed by the fact that no Schottky anomaly from isolated spins is observed in magnetic heat capacity of YbMgGaO_4_ (Fig. 3b) down to 60 mK^12^. The very low level of impurity/isolated spins also can be naturally explained by the large chemical difference between the magnetic Yb^3+^ and the other nonmagnetic ions. The difference prevents the magnetic site-mixing and hence guarantees an extremely low level of impurity/isolated spins. This is one of the key advantages of the new compound. We have made a linear fit to the magnetization data at 0.5 K in the constant susceptibility range (form 1.6 to 2.8 T) (Supplemental material), supposing it is contributed by easy-saturated “impurity” spins and field-independent “intrinsic” susceptibility^12^. The fitted intercept, ~ 0.173(4) μ_B_/Yb^3+^ (~11% of the saturation magnetization), would give ~ 11% “impurity” spins, which is an unreasonably large amount and clearly contradicts with the ESR and heat capacity measurements. The magnetization curve at 0.5 K (Fig. 4a) is very similar to that of the triangular lattice compound C_6_Eu along the *c*-axis at 4.2K^36^. In the case of C_6_Eu, this behavior has been attributed to traces of the 1/3 plateau^37^. It should be pointed out that the fitted power exponent γ also shows an unusual quasi plateau (γ ~ 1.5) in the similar magnetic-field range at extremely low temperatures (inset of Fig. 3c). The unusual χ-plateau must be related to a field-induced unconventional quantum state at extremely low temperatures (≤|θ_w_|/10).

The one-third quantum magnetization plateau, as well as higher-fraction quantum states, have been reported in Néel ordering phases (*T* < *T*_N_) in spin-1/2 triangular-lattice antiferromagnets such as Ba_3_CoSb_2_O_9_ and Cs_2_CuBr_4_, and the Néel transitions are caused by the interlayer coupling^8^,^9^,^10^. To the best of our knowledge, YbMgGaO_4_ is the first example that exhibits the abnormal magnetization behavior in a paramagnetic phase in a 2D lattice. To fully understand the abnormal magnetization behavior, further theoretical and experimental studies are required.

## Conclusion

A new triangular antiferromagnet with effective spin-1/2 and perfect 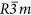 symmetry, YbMgGaO_4_, was successfully synthesized. The new compound overcomes some critical disadvantages in the existing materials, such as spatially anisotropic intralayer exchange interactions, magnetic defects, interlayer exchange coupling, and antisymmetric DM interactions. Despite the significant AF exchange coupling between neighbor spins (θ_w_ ~ −4 K), no spin freezing was observed at least down to 60 mK, suggesting that YbMgGaO_4_ is a new QSL candidate. The gapless low-energy excitations from the GS are evidenced by the low-*T* power-law temperature dependence of heat capacity and nonzero susceptibility. Almost zero residual spin entropy suggests that the new compound extremely approaches a disordered and possible gapless QSL GS at low temperatures. An unconventional field-induced quantum state featuring an abnormal susceptibility plateau was observed for the first time, where the frustrated spin system was still paramagnetic at 0.5 K.

## Methods

Yb_*x*_Lu_*1*−*x*_MgGaO_4_ (*x* = 1, 0.4, 0.16, 0.08, 0.04 and 0) white powder samples were synthesized from stoichiometric mixtures of Yb_2_O_3_ (99.99%), Lu_2_O_3_ (99.9%), MgO (99.99%) and Ga_2_O_3_ (99.999%). The mixtures were heated in air to 1450 °C for 4 days, with an intermediate grinding. The phase purities of all of the samples were confirmed by powder X-ray diffraction (XRD) (Bruker D8 Advance, 40 kV, 40 mA) before further study. High-intensity and monochromatic synchrotron XRD was used to more precisely determine the crystal structures of YbMgGaO_4_ and LuMgGaO_4_ at the diffraction station (4B9A) of the Beijing Synchrotron Radiation Facility (BSRF). The General Structure Analysis System (GSAS) program was used for Rietveld crystal structure refinements^38^. No structural transitions were observed from the XRD patterns (Rigaku, 40 kV, 200 mA) at least to 12 K in YbMgGaO_4_. Magnetization measurements at 0.48 to 300 K under 0 to 14 T were performed using a superconducting quantum interference device (SQUID) magnetometer (Quantum Design Magnetic Property Measurement System, MPMS) and a vibrating sample magnetometer (VSM) (Quantum Design Physical Property Measurement System, PPMS). The magnetizations were measured at 0.48 to 2 K using a He3 refrigeration system. The magnetizations of Yb_*x*_Lu_*1*−*x*_MgGaO_4_ (*x* = 1, 0.4, 0.16, 0.08 and 0.04) were carefully corrected for the weak contribution (<3%) of the brass sample holder to the diamagnetic background. Heat capacity measurements at 0.06 to 30 K under 0 to 9 T were performed using a Quantum Design PPMS. YbMgGaO_4_/LuMgGaO_4_ powder sample mixed with Ag (99.9%) powder (mole ratio = 1:3) was dye-pressed into hard disks to facilitate thermal equilibration. The heat capacities of YbMgGaO_4_ and LuMgGaO_4_ were carefully corrected for the separately measured Ag and other addenda (including heat-conducting glue) contributions. The heat capacity measurements between 3 K and 60 mK were performed using a dilution refrigeration system.

## Additional Information

**How to cite this article**: Li, Y. *et al.* Gapless quantum spin liquid ground state in the two-dimensional spin-1/2 triangular antiferromagnet YbMgGaO_4_. *Sci. Rep.* **5**, 16419; doi: 10.1038/srep16419 (2015).

## Supplementary Material

Supplementary Information
