# Supplementary material for: Gapless quantum spin liquid ground state in the two-dimensional spin-1/2 triangular antiferromagnet YbMgGaO4
Source: Sci Rep. 2015 Nov 10;5:16419. doi: 10.1038/srep16419 (PMC4639809; doi:10.1038/srep16419)
Supplement: Supplementary Information [file srep16419-s1.pdf]

Supplemental Material

Gapless quantum spin liquid ground state in the two-dimensional spin-1/2 triangular antiferromagnet $YbMgGaO_4$

Yuesheng Li[1], Haijun Liao[2], Zhen Zhang[3], Shiyan Li[3], Feng Jin[1], Langsheng Ling[4], Lei Zhang[4], Youming Zou[4], Li Pi[4], Zhaorong Yang[5], Junfeng Wang[6], Zhonghua Wu[7], and Qingming Zhang[*,1,8]

[1]Department of Physics, Renmin University of China, Beijing 100872, P. R. China
[2]Institute of Physics, Chinese Academy of Sciences, Beijing100190, P. R. China
[3]State Key Laboratory of Surface Physics, Department of Physics, and Laboratory of Advanced Materials, Fudan University, Shanghai 200433, P. R. China
[4]High Magnetic Field Laboratory, Chinese Academy of Sciences, Hefei 230031, P. R. China
[5]Institute of Solid State Physics, Chinese Academy of Sciences, Hefei 230031, P. R. China
[6]Wuhan National High Magnetic Field Center, Wuhan 430074, P. R. China
[7]Institute of High Energy Physics, Chinese Academy of Science, Beijing 100049, P. R. China
[8]Department of Physics and Astronomy, Collaborative Innovation Center of Advanced Microstructures, Shanghai Jiao Tong University, Shanghai 200240, P. R. China
[*]e-mail: qmzhang@ruc.edu.cn

We present here:

1. Material synthesis and measurement details
2. Synchrotron X-ray diffractions and structure refinements of $YbMgGaO_4$ and $LuMgGaO_4$
3. Low-temperature XRD patterns of $YbMgGaO_4$.
4. ESR spectra of $YbMgGaO_4$ and $Yb_{0.04}Lu_{0.96}MgGaO_4$ samples
5. Linear fit to the magnetization data of $YbMgGaO_4$ in the constant susceptibility range (form 1.6 to 2.8 T) at 0.5 K

1. Material synthesis and measurement details

$Yb_xLu_{1-x}MgGaO_4$ (x = 1, 0.4, 0.16, 0.08, 0.04 and 0) powder samples were prepared via the following chemical reaction:

$$x\mathrm{Yb_2O_3} + (1-x)\mathrm{Lu_2O_3} + 2\mathrm{MgO} + \mathrm{Ga_2O_3} = 2\mathrm{Yb}_x\mathrm{Lu}_{1-x}\mathrm{MgGaO_4}$$

Stoichiometric mixtures of high-purity Yb_2O_3 (99.99%), Lu_2O_3 (99.9%), MgO (99.99%) and Ga_2O_3 (99.999%) were ground, pressed into tablets and heated in air at 1450 °C for 4 days with an intermediate grinding step. Almost no sample mass loss was observed after heating.

To remove the influence of a possible preferred orientation of the powder samples during the crystal-structure refinements, the solid-phase, synthesized samples were carefully ground into powders with particle sizes smaller than ~ 5 μm, as determined by microscope observations. The powder samples were diluted with a moderate amount of glue, packed into capillaries (D = 0.2 mm) and prepared for X-ray diffraction using the MYTHEN detector at the diffraction station (4B9A) of the Beijing Synchrotron Radiation Facility.

For the magnetization and electron spin resonance (ESR) spectrum measurements, at least two independent powder samples were prepared and measured for each magnetic $Yb_xLu_{1-x}MgGaO_4$ composition. In addition, no clear effects caused by the possible preferred orientation of the samples were observed from the measurements.

The ESR measurements were performed using a Bruker EMX plus 10/12 CW-spectrometer at X-band frequencies (f ~ 9.4 GHz); the spectrometer was equipped with a continuous He gas-flow cryostat.

The residuals in all fits and refinements are defined as

$$R_p = \frac{\sum |y_o - y_c|}{\sum |y_o|} \quad (1)$$

where, y_o and y_c are observed and calculated values respectively. International system of units (SI) was used.

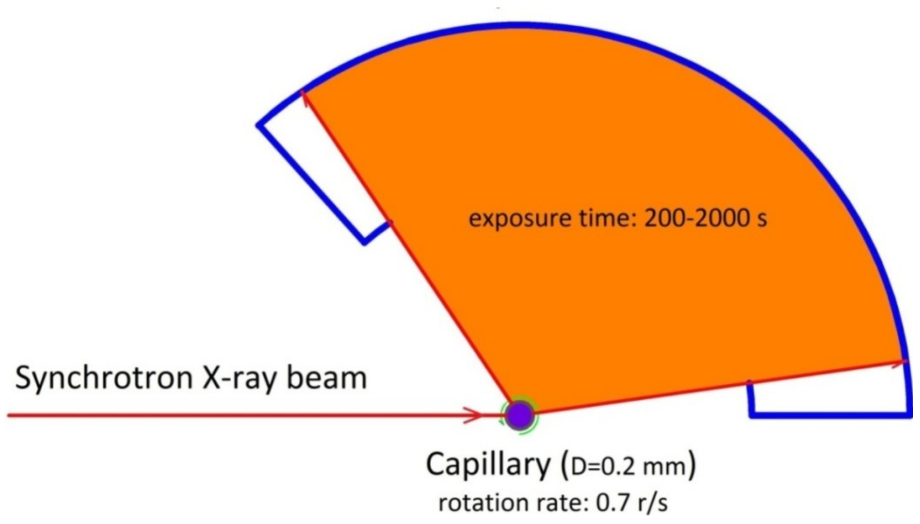

Figure S1. Diagram of the MYTHEN detector installed at the diffraction station (4B9A) of the Beijing Synchrotron Radiation Facility.

2. Synchrotron X-ray diffractions and structure refinements of $YbMgGaO_4$ and $LuMgGaO_4$

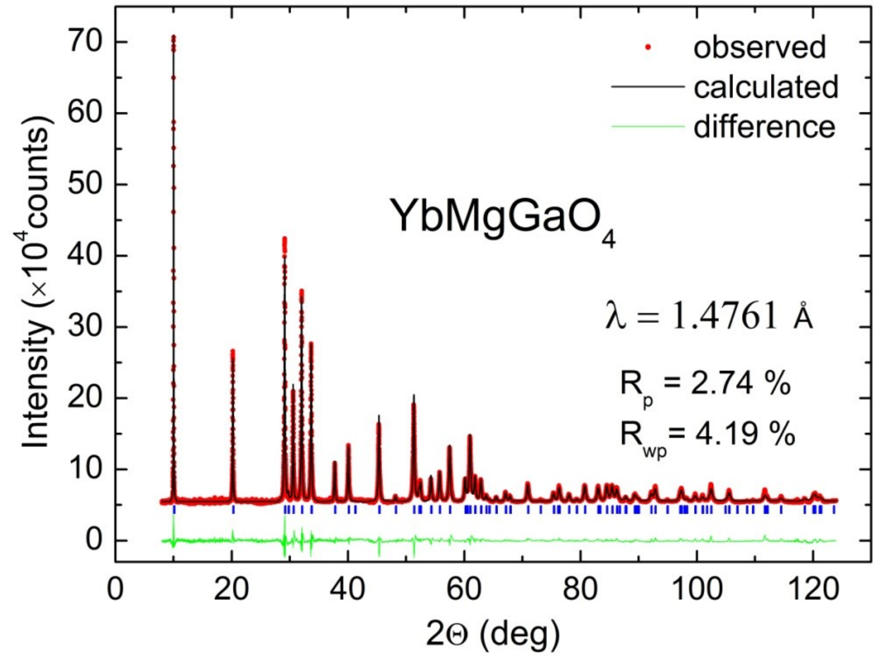

Figure S2. Synchrotron X-ray diffraction and final Rietveld refinement profiles for $YbMgGaO_4$ at 300 K.

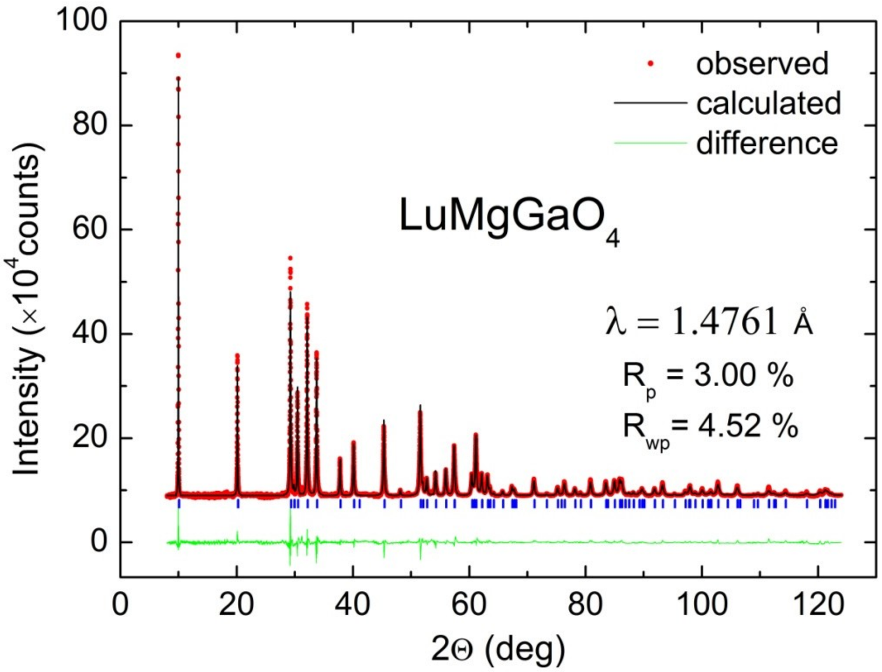

Figure S3. Synchrotron X-ray diffraction and final Rietveld refinement profiles for $LuMgGaO_4$ at 300 K.

Table S1. Refined crystal structures of $YbMgGaO_4$ and $LuMgGaO_4$

Compound		$YbMgGaO_4$		$LuMgGaO_4$	
Model		**I**	**II**	**I**	**II**
Space group		$R\bar{3}m$			
Lattice	a (Å)	3.40212(8)	3.40282(6)	3.38750(5)	3.38750(5)
	c (Å)	25.1191(6)	25.1243(5)	25.2069(4)	25.2071(4)
Yb/Lu^{3+}	Fraction	1	0.5	1	0.5
	z	0	0.00439(6)	0	0.00425(6)
	Uiso (×100)	1.016(18)	0.751(21)	0.981(18)	0.637(20)
Mg^{2+}/Ga^{3+}	Fraction	0.5	0.5	0.5	0.5
	z	0.214902(29)	0.214918(29)	0.215397(30)	0.215385(30)
	Uiso (×100)	0.216(26)	0.301(26)	0.332(27)	0.226(27)
$O1^{-2}$	z	0.29260(10)	0.29221(10)	0.29177(11)	0.29213(11)
	Uiso (×100)	0.92(8)	0.72(8)	1.15(8)	1.04(8)
$O2^{-2}$	z	0.12967(9)	0.12993(9)	0.12967(10)	0.12971(10)
	Uiso (×100)	0.54(8)	0.47(7)	1.02(8)	0.68(7)
Residuals	R_{wp}	0.0419	0.0412	0.0452	0.0442
	R_p	0.0274	0.0268	0.0300	0.0292

3. Low-temperature XRD patterns of $YbMgGaO_4$.

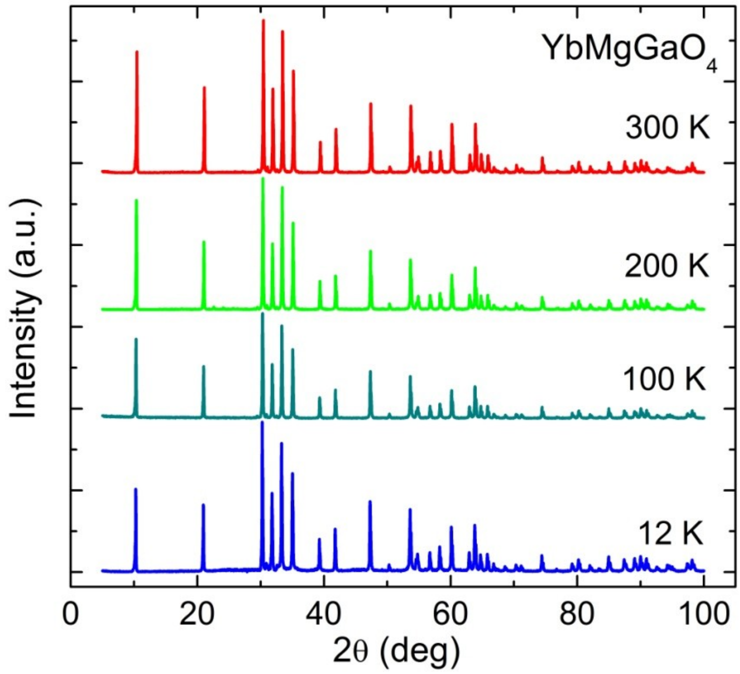

Figure S4. X-ray (Cu-K_α) diffraction profiles for $YbMgGaO_4$ at 300, 200, 100 and 12 K respectively. No additional reflections are observed at temperatures down to 12 K, indicating that no structural transitions occurred.

4. ESR spectra of $YbMgGaO_4$ and $Yb_{0.04}Lu_{0.96}MgGaO_4$ samples

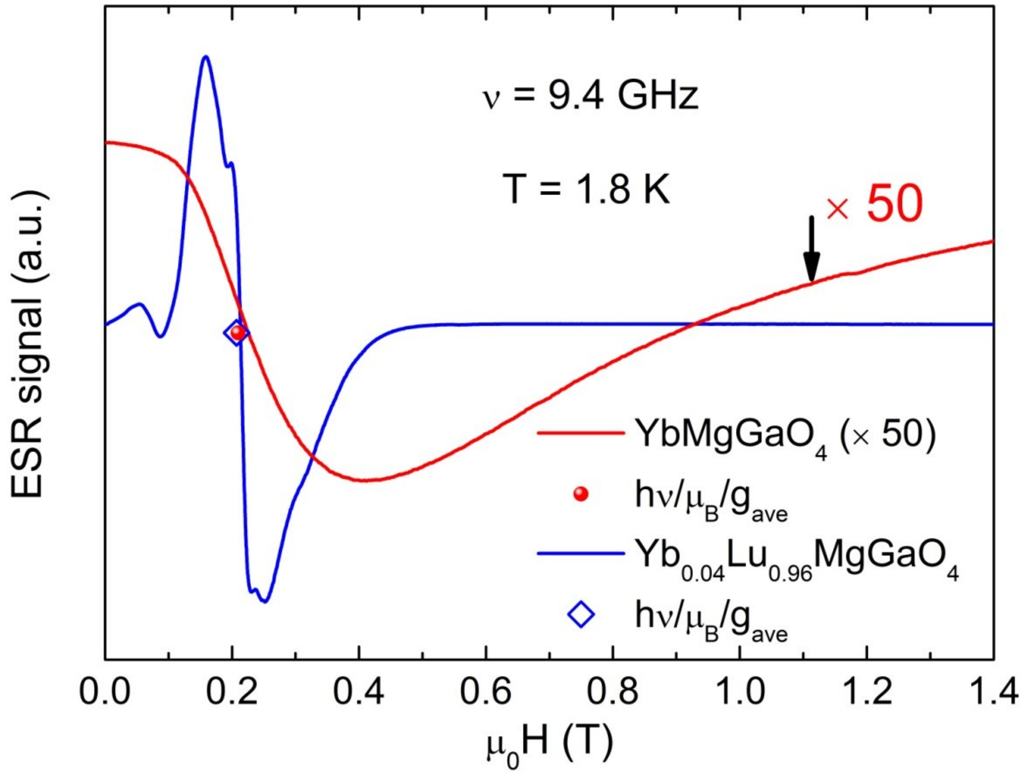

Figure S5. X-band (9.4 GHz) Yb^{3+} first-derivative absorption ESR spectra for $YbMgGaO_4$ and $Yb_{0.04}Lu_{0.96}MgGaO_4$. The narrow and intense hyperfine lines[1] observed in the quasi-free spin compound $Yb_{0.04}Lu_{0.96}MgGaO_4$ completely disappear in the spectrum of $YbMgGaO_4$, suggesting that no observable isolated Yb^{3+} or magnetic defects (< 0.04 %) are present in $YbMgGaO_4$.

5. **Linear fit to the magnetization data of $YbMgGaO_4$ in the constant susceptibility range (form 1.6 to 2.8 T) at 0.5 K**

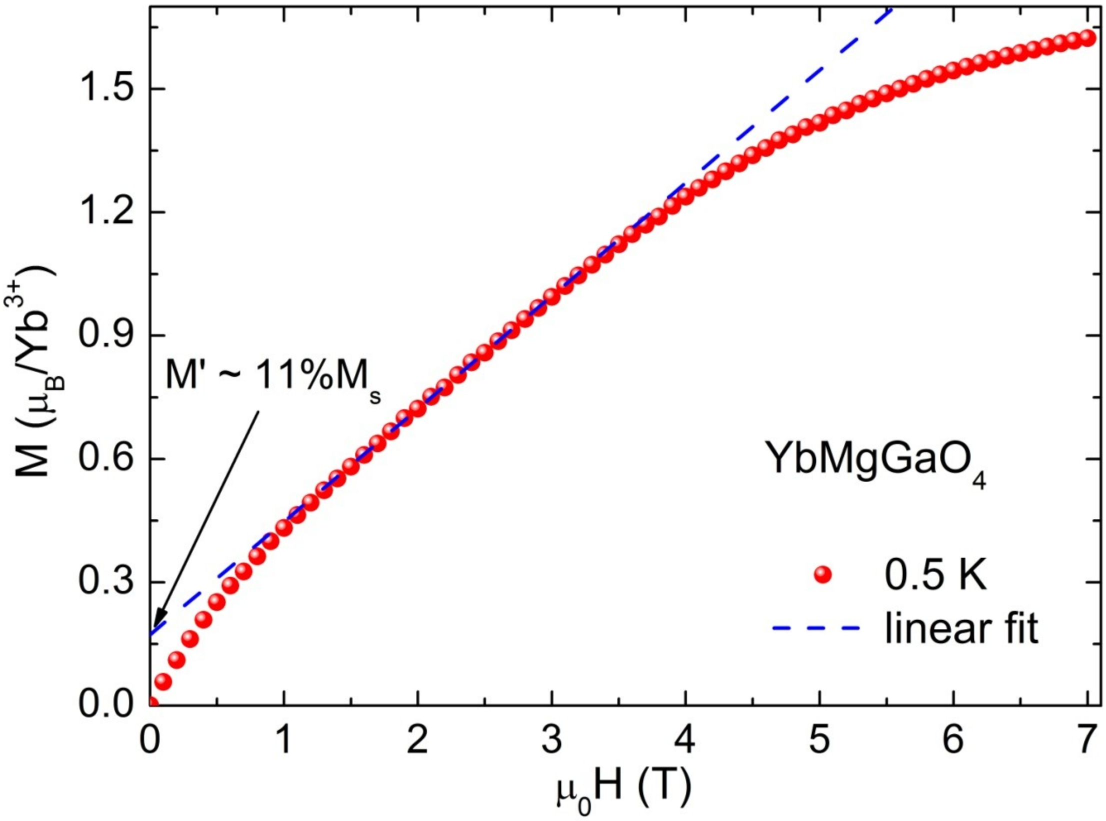

Figure S6. Linear fit to the magnetization data of $YbMgGaO_4$ in the constant susceptibility range (form 1.6 to 2.8 T) at 0.5 K. The fitted intercept, ~ 0.173(4) μ_B/Yb^{3+} (the convex magnetization), gives ~ 11% of the saturation magnetization.

References

1. Misra, S. K. & Isber, S. EPR of the Kramers ions Er^{3+}, Nd^{3+}, Yb^{3+} and Ce^{3+} in $Y(NO_3)_3 \cdot 6H_2O$ and $Y_2(SO_4)_3 \cdot 8H_2O$ single crystals: Study of hyperfine transitions. *Physica B* **253**, 111-122 (1998).
